# Food Odours Direct Specific Appetite

**DOI:** 10.3390/foods5010012

**Published:** 2016-02-22

**Authors:** Harriët F. A. Zoon, Cees de Graaf, Sanne Boesveldt

**Affiliations:** Division of Human Nutrition, Wageningen University, P.O. Box 8129, 6700 EV Wageningen, The Netherlands; kees.degraaf@wur.nl (C.G.); sanne.boesveldt@wur.nl (S.B.)

**Keywords:** sensory-specific appetite, olfaction, taste, energy density

## Abstract

Olfactory food cues were found to increase appetite for products similar in taste. We aimed to replicate this phenomenon for taste (sweet/savoury), determine whether it extends to energy density (high/low) as well, and uncover whether this effect is modulated by hunger state. Twenty-nine healthy-weight females smelled four odours differing in the energy density and taste they signalled, one non-food odour, and one odourless solution (control), in random order, for three minutes each. Appetite for 15 food products was rated in the following two minutes. Mixed model analyses revealed that exposure to an odour signalling a specific taste (respectively sweet, savoury) led to a greater appetite for congruent food products (sweet/savoury) compared to incongruent food products (savoury *p* < 0.001; sweet *p* < 0.001) or neutral food products (*p* = 0.02; *p* = 0.003). A similar pattern was present for the energy-density category (respectively high-energy dense, low-energy dense) signalled by the odours (low-energy products *p* < 0.001; high-energy products *p* = 0.008). Hunger state did not have a significant impact on sensory-specific appetite. These results suggest that exposure to food odours increases appetite for congruent products, in terms of both taste and energy density, irrespective of hunger state. We speculate that food odours steer towards intake of products with a congruent macronutrient composition.

## 1. Introduction

Olfactory cues of palatable food appear to work as appetizers while anticipating food intake. The smell of freshly baked bread entices you to buy and eventually eat a loaf.

From the day we are born we experience food odours during anticipation and consumption of food. Consumption is followed by nutritional consequences related to satiety, such as the digestion of available macronutrients. Over the course of our life we learn to use sensory food cues to (accurately) anticipate the energy density and taste (sweet/savoury) of the foods we are about to eat [1,2]. Through cephalic phase responses, our body is able to prepare for what will be ingested [3,4,5]. Food odours thus are an important guide in our food-rich environment, but their exact role needs to be clarified.

Olfactory food cues presented in the anticipatory phase of eating are found to increase the appetite for congruent products and decrease the appetite for incongruent products [6,7]. This phenomenon is referred to as sensory-specific appetite. Besides product-specific effects, Ramaekers *et al.* [6,7] found that savoury odours increased the appetite for (other) savoury foods and decreased the appetite for sweet foods, and *vice versa*. In earlier research, exposure to food odours (pizza, cookies) increased appetite, liking and craving for the food that was smelled in restrained eaters [8,9]. Moreover, Gaillet *et al.* [10,11] found that non-attentively perceived odours in the environment affected food choice. Participants placed in a waiting room with pear odour, chose fruity desserts more often compared to participants that had been waiting in an unscented room. Brief exposure to the smell and sight of pizza increased prospective intake for pizza and other savoury foods, but not for sweet foods [12,13]. After pizza cueing, the amount of pizza participants thought they could eat accurately predicted how much pizza they would actually eat [12]. Fedoroff *et al.* [8,9] and Larsen *et al.* [14] also found that participants ate more of an odour-cued food compared to non-cued food. Increased appetite likely parallels the effects of odour exposure on food choice, prospective intake and actual intake for matching products (*i.e*, sweet/savoury, similar energy density, fruity).

The results of Ramaekers *et al.* [6,7] indicate the presence of sensory-specific appetite for taste category (sweet *vs*. savoury). Taste is important in the prediction of macronutrient content of a food. Savoury tastes are thought to indicate a high-protein content, whereas sweet tastes are suggested to point to a high-carbohydrate content, e.g., [15,16,17]. Energy density of a food, such as fat content, is ecologically relevant as well. It has been suggested that humans are able to detect fat content of a food using their sense of smell [18]. Anticipation of the energy density of a food is important in the process of energy-intake regulation.

Internal cues of hunger and satiety impact how much we eat [19,20]. It is likely that these internal cues also play a modulating role in food-cue reactivity during the anticipation phase of eating and thereby influence our appetite (responses) and drive to ingest energy.

In this study we aim to replicate the influence of olfactory cues on sensory-specific appetite for a certain taste category and extend those findings to energy-density categories of foods. Additionally, we are interested whether hunger state plays a modulatory role in this effect. We expect that for both taste and energy density, exposure to food odours will lead to an increased appetite for products that are congruent to the odour and a decreased appetite for products that are incongruent to the odour. Additionally, we hypothesize that sensory-specific effects on appetite are thought to be more relevant and thus more pronounced in a hungry compared to a satiated state.

## 2. Methods

### 2.1. Overall Design

This study followed a 2 × 6 within-subjects design, including the factors hunger state (hungry/satiated) and odour category (high-energy sweet: HESw, high-energy savoury: HESav, low-energy sweet: LESw, low-energy savoury: LESav, non-food control: NF, and no-odour: Baseline). We aimed to determine olfactory sensory-specific appetite for the taste and energy-density categories and assess whether hunger state modulates these effects.

### 2.2. Participants

Twenty-nine healthy-weight females (Body Mass Index (BMI): 21.3 ± 1.4 kg/m^2^; age: 27 ± 11 years) participated in this study (see demographics in Table 1). To ensure that all participants were normal weight (BMI: 18.5–25 kg/m^2^), body weight (kg) and length (m) were determined. Further, participants had to be normosmic (scoring ≥12 on the Sniffin’ Sticks 16-item identification test; [21]), in general good health (subjective), not using medication other than paracetamol and oral contraceptives, and had to be weight stable for at least two months. Restraint score (1–5) was determined by using the restraint subscale of the Dutch Eating Behaviour Questionnaire (DEBQ; [22]). Individuals scoring higher than 2.8 on dietary restraint were excluded from participation. Respondents that did not like the products used in the study (<40 mm on a 100 mm Visual Analogue Scale (VAS)) were excluded. We also did not include participants that had a smoking habit, had convictions that restricted consumption of certain products (vegetarian, vegan, not eating beef, *etc*.) or had mental or physical status that could hinder the study procedures (e.g., food allergy, endocrine abnormality). Participants received a voucher of €25 for their contribution. All participants provided written informed consent before they participated in the study. This study was conducted in accordance with the Declaration of Helsinki of 1975, revised in 2013. The protocol was approved by the Medical Ethical Committee of Wageningen University (NL46034.081.13).

### 2.3. Experimental Procedure

Participants attended two separate test sessions, once in a hungry state and once in a satiated state. For the hungry condition participants were asked to refrain from eating and drinking anything but water and weak tea in the three hours before the test session. For the satiated condition participants were asked to consume a comfortably satiating meal within the hour, but minimally 30 min before the test session. They were instructed to drink 0.5 L water 30 min before both test sessions to prevent dehydration. The order of the hunger state conditions was counterbalanced over participants. At the beginning of each test session participants rated their hunger (hunger, fullness, prospective consumption, desire to eat, and thirst) on a 100 mm VAS (EyeQuestion, Version 3.11.1, Logic8 BV, Elst, The Netherlands).

Every test session, participants were provided with six brown 50 mL bottles, each containing a different solution (5 mL). Participants were instructed to smell each bottle for three minutes. A short break of two minutes was used between odour exposures to avoid carry-over effects of the previous odour exposure [23]. In this time participants rated odour intensity and general appetite (100 mm VAS). Subsequently, participants indicated their appetite for 15 products belonging to five different categories (HESw, HESav, LESw, LESav, neutral control). The order in which the odours were presented was randomized between participants. Each participant received the same order of odours in both test sessions.

### 2.4. Olfactory Stimuli

We performed a pilot study in a separate sample of participants (*n* = 15), to select odours signalling either HESw food, HESav food, LESw food, LESav food, NF as control and a no-odour solution as baseline reference. Odour selection was based on similarities in perceived intensity and liking ratings, differences in the associated energy-density and taste categories, and consistent matching of the odour to a product/object. The selected odours included chocolate (HESw; International Flavors and Fragrances (IFF) 10810180; 5% in Propylene Glycol (PG)), beef (HESav; IFF 10878095; 0.04% in demi water), melon (LESw; IFF 15025874; 20% in PG), cucumber (LESav; IFF 73519595; 100%), fresh green (NF; AllSens–Voit Aroma Factory No. 819; 1% in PG), no-odour (baseline reference, 100% propylene glycol). Liking and familiarity ratings given by the 29 participants during a screening procedure can be found in the supplemental materials (Table S1). Odour intensity rated during the test sessions can also be found in the supplementary materials (Table S2).

### 2.5. Products Appetite Questionnaire

Based on results of a second pilot study (*n* = 17), 15 food products were selected for the appetite questionnaire. The selection was based on ratings of liking, estimated caloric content, and on the indicated category (HESw, HESav, LESw, LESav, neutral) of the product. All of the selected products can be considered as snack foods. Three products were included for each category (HESw, HESav, LESw, LESav) and three neutral food products (in terms of taste) were added as control. HESw products included pieces of chocolate, cake and stroopwafel (a Dutch caramel syrup waffle); for HESav we selected beef croquette, cheese cubes and crisps; the LESw products were a slice of melon, an apple and strawberries; for the LESav products we selected a piece of cucumber, tomato salad and raw carrot; bread, croissants and pancake were included as neutral controls. Liking and familiarity ratings for the products given by the 29 participants during a screening procedure are presented in Table S3.

### 2.6. Data Analyses

#### 2.6.1. Hunger State

A paired samples *T*-test was performed on the hunger ratings (*i.e.*, hunger, fullness, prospective consumption, desire to eat, and thirst) to confirm that participants were in different hunger states in the two test sessions they attended.

#### 2.6.2. Appetite Ratings

All main analyses were performed following a linear mixed effects models procedure in IBM SPSS Statistics for Windows, Version 22.0 (IBM Corp., Armonk, NY, USA). First-order ante-dependence was chosen as covariance structure. A *p*-value of <0.05 was considered significant. Session order and participant number were added as repeated variables. Hunger state (hungry, satiated) was included as covariate. Differences in rated odour intensity between the odours was tested with a mixed model including odour category (HESw, HESav, LESw, LESav, NF, Baseline) as fixed effects factor.

General appetite ratings (100 mm VAS) were analysed by adding raw ratings of general appetite as dependent factor and odour category (HESw, HESav, LESw, LESav, NF, Baseline) as fixed effects factor.

We were interested in specific appetite effects induced by odorants signalling different categories. We used appetite ratings after exposure to the no-odour solution as a baseline reference, and subtracted these appetite ratings from ratings provided after smelling an odour, yielding Δ appetite scores. To determine sensory-specific effects of taste category, data were split by taste category (Sav, Sw, NF odours). Products were then pooled per taste category. Δ Appetite was used as outcome measure. Product taste category was added as fixed effects factor. Similarly, sensory-specific effects of the energy-density category were analysed by distinguishing the data by energy category (HE, LE odours). Products were then pooled per energy category. We added Δ appetite as outcome measure and product energy category as fixed effects factor. *Post-hoc* paired comparisons were used to uncover effects of the odours on the separate product categories. To exclude the possibility that the effects were merely driven by the specific odour-product match (e.g., chocolate odour exposure = appetite for chocolate pieces), the analyses described above were rerun without the specific matches.

## 3. Results

### 3.1. Hunger State (Manipulation Check)

Hunger ratings confirmed that feelings of hunger were significantly different between sessions, and according to the appropriate hunger states (hungry *vs*. satiated; see Table 2). In the hungry conditions the participants felt hungrier, less full, and indicated a higher prospective consumption and desire to eat (all *p* < 0.001). Ratings of thirst indicated that participants were equally thirsty in both test sessions.

### 3.2. General Appetite

General appetite (100 mm VAS) differed significantly after exposure to different odours (*p* = 0.015) and also between hunger states (*p* < 0.001). General appetite after smelling chocolate (46 ± 3), beef (42 ± 4), melon (44 ± 3) and cucumber (47 ± 3) was significantly higher than after smelling fresh green (36 ± 3) or baseline reference (36 ± 3; all *p* < 0.01). General appetite in the hungry state (62 ± 3) was significantly higher compared to the satiated state (21 ± 3; *p* < 0.001).

### 3.3. Sensory-Specific Appetite (SSA): Taste Category

**Figure 1:**
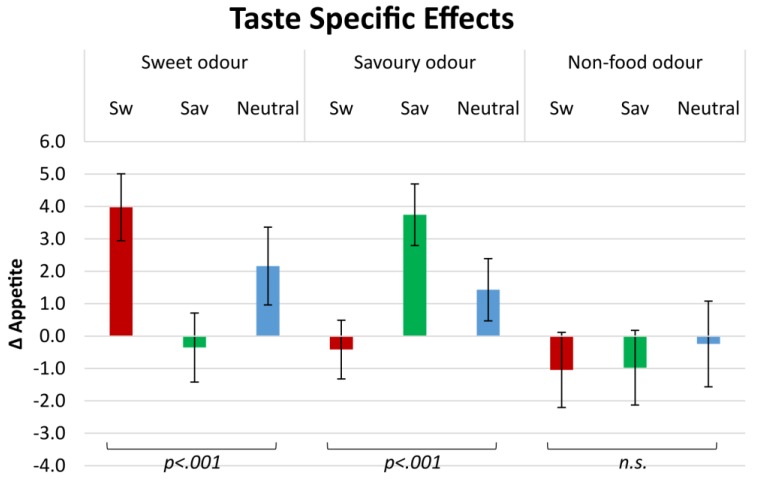
Δ Appetite (on 100 mm VAS; appetite after smelling an odour minus appetite after smelling a baseline reference) for sweet (Sw), savoury (Sav) and neutral products after smelling sweet, savoury and non-food odours.

Our results (see Figure 1) show that by smelling Sw odours, appetite changes significantly for products of different taste categories (*F*(2, 432) = 27.46, *p* < 0.001). After exposure to Sw odours, Δ appetite was significantly higher for Sw products (4.0 ± 1.1) than for Sav (−0.4 ± 1.1; *p* < 0.001) or neutral products (2.2 ± 1.2; *p* = 0.022), and Δ appetite for Sav products was also significantly lower than appetite for neutral products (*p* = 0.001).

Similarly, Δ appetite after smelling Sav odours was significantly different between the product-taste categories (*F*(2, 404) = 12.08, *p* < 0.001). It was higher for Sav products (3.7 ± 1.2) than for Sw (−0.4 ± 1.3; *p* < 0.001) or neutral products (1.4 ± 1.3; *p* = 0.003), but did not differ between Sw and neutral products (*p* = 0.017).

Δ Appetite after smelling an NF odour was not significantly different for the different products (*F*(2, 197) = 0.28, *p* = 0.750). For Sw products (−1.0 ± 0.9), Sav products (−1.0 ± 0.9) or neutral products (−0.2 ± 0.9; all *p* > 0.05).

In addition to an increase in general appetite when hungry (Section 3.2), participants’ raw appetite ratings for products were higher in the hungry state (see Figure S1). However, there were no significant differences in specific appetite (the difference between appetite after exposure to the no-odour control and appetite rated after exposure to an odour) between the hungry and satiated condition (difference in Δ appetite between hunger states after Sw odour: 0.7 mm; after Sav odour: 0.0 mm; after NF odour: 2.6 mm; all *p* > 0.05).

In order to exclude the possibility that the effects described above were driven by the specific odour-product match (e.g., exposure to chocolate odour = appetite for chocolate pieces), these specific matches were excluded from the dataset and the analyses were rerun.

We found a significant main effect of Sw odour exposure on Δ appetite for different product taste categories (*F*(2, 403) = 19.29, *p* < 0.001). Δ Appetite after Sw odours remained significantly higher for Sw products (3.1 ± 1.1) than for Sav products (−0.4 ± 1.1; *p* < 0.001), but did not differ from Δ appetite for neutral products (2.0 ± 1.1; *p* = 0.191.). Further, Δ appetite for Sav products also differed from Δ appetite for neutral products (*p* = 0.003).

Δ Appetite after smelling Sav odours was also significantly different between product categories that were based on taste (*F*(2, 383) = 5.68, *p* = 0.004). Δ appetite for Sav products (2.8 ± 1.3) remained significantly higher than for Sw products (−0.4 ± 1.3; *p* < 0.001), but was not significantly different from Δ appetite for neutral products (1.2 ± 1.3; *p* = 0.052). Δ Appetite for Sw products was significantly different from Δ appetite for neutral products (*p* = 0.033).

### 3.4. SSA: Energy Category

**Figure 2:**
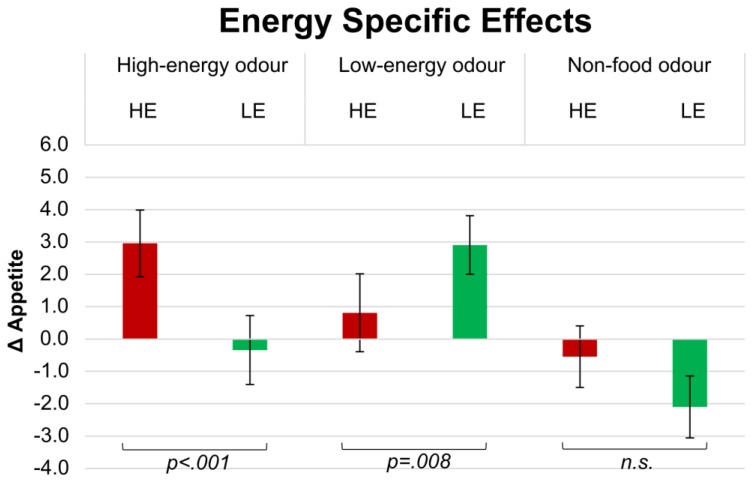
Δ Appetite (appetite after smelling an odour minus appetite after smelling a baseline reference) for high-energy (HE), low-energy (LE) products after exposure to high-energy (HE), low-energy (LE) and non-food (NF) odours.

After smelling HE odours, Δ appetite for HE products (3.0 ± 1.1) was significantly higher than for LE (−0.3 ± 1.2; *p* < 0.001; see Figure 2).

Paired comparisons also revealed that Δ appetite rated after smelling LE odours was significantly higher for LE products (2.9 ± 1.2) than for HE products (0.8 ± 1.1; *p* = 0.008).

Δ Appetite rated after smelling an NF odour was not significantly different than for HE products (−0.5 ± 0.9) and LE products (−2.1 ± 1.1).

In Figure S2 it is visible that raw appetite ratings for products are higher in the hungry state. Specific appetite (the difference between appetite after exposure to the no-odour control and appetite rated after exposure to an odour) was not significantly influenced by hunger state (difference in Δ appetite between hunger states after HE odour: 0.7 mm; after LE odour: 0.0 mm; both *p* > 0.05).

As mentioned in section 3.3, the analyses were rerun, excluding specific matches from the dataset, to account for the possibility that the effects described above were merely driven by the specific odour-product match.

Paired comparisons showed that after smelling HE odours, Δ appetite for HE products (2.3 ± 1.1) was significantly different from Δ appetite for LE products (−0.7 ± 1.1; *p* < 0.001).

Δ Appetite after smelling LE products was not different for LE products (1.8 ± 1.2) and HE products (0.6 ± 1.1).

## 4. Discussion

With this research, we wanted to determine whether odours signalling specific categories (sweet/savoury taste; high-/low-energy density; non-food) can induce sensory-specific changes in appetite. Furthermore, we were interested in whether the strength of this effect was dependent on hunger state. Results reveal that food odours increase appetite for products that are similar, both in taste and energy density. Hunger state did not significantly affect odour-induced sensory-specific appetite.

Our results indicate that odours that signal sweet products and odours that signal savoury products affect appetite in an opposite, but similar way. After smelling a sweet food odour, appetite for sweet products increases more compared to appetite for neutral (bread, croissant, and pancake) and savoury products. Similarly, after smelling a savoury food odour, appetite for savoury products increases significantly more than for neutral or sweet products. In line with this, the smell of flowers (fresh green), a non-food related odour, slightly decreased appetite ratings for sweet, savoury and neutral foods equally. These results are concurrent with those of Ramaekers *et al.* [6,7]. We suggest that the level of congruency between the odour and the product determines the strength of the appetizing effect of the odours. Interestingly, after removing the specific odour-product matches (e.g., chocolate odour = appetite for chocolate pieces) from our dataset, appetite for taste-congruent products remained higher compared to non-congruent products. Olfactory sensory-specific appetite is a phenomenon that not only applies to a specific product, but also to categories of products related to taste quality (sweet *vs*. savoury). By repeated exposure to the combination of an odour and the nutritional consequences after eating the food we smell, it is proposed that we learn to use (olfactory) food cues as predictors for macronutrient content [1,2]. These associations are most often described in terms of taste quality. Sweet taste is related to a high-carbohydrate content, whilst savoury taste is associated with a high-protein content [15,16,24].

To our knowledge, this is the first study investigating olfactory sensory-specific appetite effects for energy density (high-energy *vs*. low-energy). When odours and products were categorized according to their associated energy density, we saw that appetite changed in a similar pattern as for taste category. Smelling odours associated with high-energy dense food increased the appetite for high-energy products significantly more than for low-energy dense products. The reverse occurred after smelling odours of low-energy dense foods. There was no difference in appetite for high-energy dense and low-energy dense products after smelling a non-food related odour. After removing the specific odour-product matches from the dataset, the pattern remained the same for high-energy dense odours. However, low-energy odours did not induce category-specific appetite effects. High-energy dense odours appear to be more potent appetizers than low-energy dense odours. Sensory-specific appetite seems to exist for specific odour-product matches and for the high energy-density category more in general.

When comparing these effects to the effects of the taste category, it seems that olfactory signals of taste lead to larger increases in appetite than for olfactory signals of energy density. After removal of the specific odour-product matches, an interesting pattern in the results emerges. Category-broad effects for sweet, savoury and high-energy dense odours remain, whereas a significant category-broad effect for low-energy food odours is absent. Based on these results, we speculate that odours signal macronutrient content. Low-energy food cues signal products that do not have substantial content. This can explain the lack of category-broad effect for low-energy cues. As mentioned for the taste category, it is thought that sweet signals carbohydrates (sweet) and savoury signals protein [15,16,24]. On the contrary, the energy density of a food is not based on a single macronutrient *per se*, but can be composed of different macronutrient sources, mainly fats and carbohydrates. In essence, teasing apart energy and macronutrient contribution is complex since they are interrelated. A systematic and more complete research approach is necessary to uncover the appetizing effects of olfactory stimuli representing proteins, carbohydrates and fats. Deprivation or supplementation of a certain macronutrient by an experimental diet could unveil whether appetizing effects of odours are modulated by macronutrient status. This will help to determine whether the function of macronutrient signalling can indeed be ascribed to olfactory cues in the anticipatory phase of eating.

Intake regulation is largely dependent on feelings of hunger and satiation [25,26]. Individuals that maintain their energy balance should be able to adjust their food intake according to their hunger status and resist eating beyond satiety when tempted by environmental food cues [8]. Hunger ratings provided by the participants in our study confirmed that feelings of hunger (hunger, fullness, prospective consumption, desire to eat) were significantly different in the two hunger states (hungry *vs*. satiated; see Table 2). Surprisingly, hunger state did not modulate odour-induced specific appetite: Δ Appetite for specific products after odour exposure was no different when participants were satiated or not. The theory that tempting food cues in the environment stimulate eating in the absence of hunger has been posed before [27]. This idea is supported by research by Nijs *et al.* [28], where participants showed a similar bias in orientation and maintained attention to visual food cues in both a hungry and satiated condition. Appetite responses of our participants also show that the impact of food odour exposure is independent of hunger state.

Selective detection of nutritious foods has been a beneficial trait for survival throughout evolution. We propose that in the anticipation phase of eating, (olfactory) food cues induce appetite specific to the macronutrient content that is signalled by the odour. Besides changes in appetite behaviour [8,9], previous research revealed very rapid physiological changes (e.g., salivation, endocrine responses; [4,29]) following food odour exposure. It is not clear whether food cues signalling a specific macronutrient content cause specific physiological responses to facilitate macronutrient uptake. Over the course of evolution, it would seem advantageous to have built-in systems (physiological, behavioural) that work together to obtain food sources in the environment.

Although olfactory food cues in our surroundings (ambient exposure) clearly have a specific effect on appetite, the effect on food preferences or choice is less consistent. Food odours do not seem to affect the preference for one type of food over another, as measured by a forced choice computer task [19], but do influence what we decide to eat when choosing dishes from a menu [10,11]. These inconsistencies in choice are likely related to variations in context (controlled *vs.* real-life). Ferriday and Brunstrom [12,13] showed that smelling and viewing pizza increases the amount of pizza we think we can eat (prospective consumption), a measure closely related to appetite. This effect also transferred to dishes that were similar to pizza (“scrambled egg, chips and baked beans”, “pasta and tomato sauce”), but not to dishes that were dissimilar (cake).What is more, after smelling a food (in combination with the sight), participants ate more of the cued food [8,9,12,14]. However, findings on intake are not consistently repeated [19] and seem to have specific prerequisites with regard to the context, cue exposure and also personality characteristics (restraint, impulsivity). Altogether, it seems that olfactory food cues have a more clear-cut role in the phase leading up to meal initiation [30], tempting us to start eating what is in front of us. Their influence may wane in later phases, when other cues such as flavour (taste, retronasal odour), satiety signals and intake-inhibiting behaviours come into play, that work toward cessation of eating. In the context of food scarcity and low food security, it is highly beneficial to have the ability to detect nutritious food sources. Subsequent increases in appetite and changes in physiology may promote and facilitate adequate food intake and uptake, thereby increasing chances of survival. In most Western societies, however, energy-dense foods are readily available. In this case, appetizing olfactory food cues are part of an environment that promotes overconsumption, ultimately contributing to a higher incidence of nutrition-related diseases (e.g., obesity, diabetes).

We cannot exclude that our sample size was too small to detect modulatory effects of hunger state on specific appetite. However, we do not find this plausible since significant effects of hunger state on general appetite were found. Confirmation of the absence of this effect may be provided by future research in a larger study population. Additionally, in the current study we only included females, limiting the possibility to generalize our findings to a broader population that includes males.

## 5. Conclusions

In conclusion, we show that food odours increase appetite for products with a similar taste and/or energy density. Odours thus steer towards intake of congruent food products and perhaps help to prepare the body for digestion of this product. This process seems to rely more on taste quality (related to macronutrient content) than on the amount of calories a food provides (energy density). We propose that food odours in the anticipatory phase of eating transfer information about the macronutrient content of the food. Moreover, food odours appear to elicit their appetizing effects in both hungry and satiated states, indicating that exposure to food odours can promote overeating and could contribute to obesity. Conversely, these effects may be used to stimulate appetite and meal initiation in people that are undernourished.

## Figures and Tables

**Table 1:** Population (*N* = 29) description by demographic and personality characteristics.

Characteristic	Mean ± SD
Age (years)	27.2 ± 11.5
BMI (kg/m^2^)	21.3 ± 1.4
Olfactory performance (Sniffin’ Sticks Identification 16)	13.6 ± 1.3
DEBQ: Restrained	2.2 ± 0.4

**Table 2:** Hunger ratings (100 mm VAS) for both hunger states.

Parameter	*Hungry (Mean ± SE)*	*Satiated (Mean ± SE)*
Hunger ***	58 ± 4	11 ± 2
Fullness ***	22 ± 4	65 ± 3
Prospective consumption ***	61 ± 3	29 ± 4
Desire to eat ***	65 ± 4	18 ± 2
Thirst	38 ± 6	27 ± 4

*** p-Value < 0.001, paired samples *T*-test.

## Supplementary Files

Supplementary File 1
