# Supplementary material for: Food Odours Direct Specific Appetite
Source: Foods. 2016 Feb 22;5(1):12. doi: 10.3390/foods5010012 (PMC5224573; doi:10.3390/foods5010012)
Supplement: Supplementary File 1 [file foods-05-00012-s001.docx]

Food Odours Direct Specific Appetite

Harriët F. A. Zoon *, Cees de Graaf and Sanne Boesveldt

Supplementary materials

**Table S1.** Liking and familiarity ratings (100 mm VAS) of the odours that were used in the test sessions. Ratings for all odours >40 mm.

| **Odour** | **Category** | **Liking (Mean ± SE)** | **Familiarity (Mean ± SE)** |
| --- | --- | --- | --- |
| Chocolate | High-energy: Sweet | 75 ± 21 | 82 ± 22 |
| Beef | High-energy: Savoury | 53 ± 27 | 68 ± 29 |
| Melon | Low-energy: Sweet | 65 ± 26 | 76 ± 23 |
| Cucumber | Low-energy: Savoury | 64 ± 20 | 81 ± 22 |
| Fresh Green | Non-food control | 63 ± 22 | 64 ± 24 |
| No-Odour | Baseline reference | 49 ± 16 | 35 ± 22 |

**Table S2.** Average odour intensity ratings (100 mm VAS) after 3 min of smelling.

| **Odour** | ***(Mean ± SE)*** |
| --- | --- |
| Chocolate | 75 ± 3 |
| Beef | 74 ± 3 |
| Melon | 73 ± 2 |
| Cucumber | 58 ± 3 |
| Fresh Green | 76 ± 4 |
| No-Odour | 7 ± 2 |

**Table S3.** Liking and familiarity ratings (100 mm VAS) of the products that were used in the appetite questionnaire.

| **Product** | **Category** | **Liking (Mean ± SE)** | **Familiarity (Mean±SE)** |
| --- | --- | --- | --- |
| Pieces of chocolate | High-energy: Sweet | 93 ± 9 | 97 ± 4 |
| Cake |  | 84 ± 12 | 94 ± 8 |
| Stroopwafel |  | 84 ± 16 | 92 ± 12 |
| Beef croquette | High-energy: Savoury | 81 ± 15 | 86 ± 14 |
| Cheese cubes |  | 81 ± 15 | 91 ± 14 |
| Crisps |  | 88 ± 11 | 96 ± 7 |
| Slice of melon | Low-energy: Sweet | 90 ± 11 | 92 ± 9 |
| Apple |  | 84 ± 13 | 96 ± 8 |
| Strawberries |  | 94 ± 11 | 96 ± 6 |
| Piece of cucumber | Low-energy: Savoury | 81 ± 13 | 97 ± 4 |
| Tomato salad |  | 80 ± 14 | 81 ± 20 |
| Raw carrot |  | 76 ± 19 | 92 ± 11 |
| Bread | Neutral control | 80 ± 15 | 97 ± 5 |
| Croissant |  | 79 ± 14 | 92 ± 10 |
| Pancake |  | 87 ± 10 | 95 ± 6 |


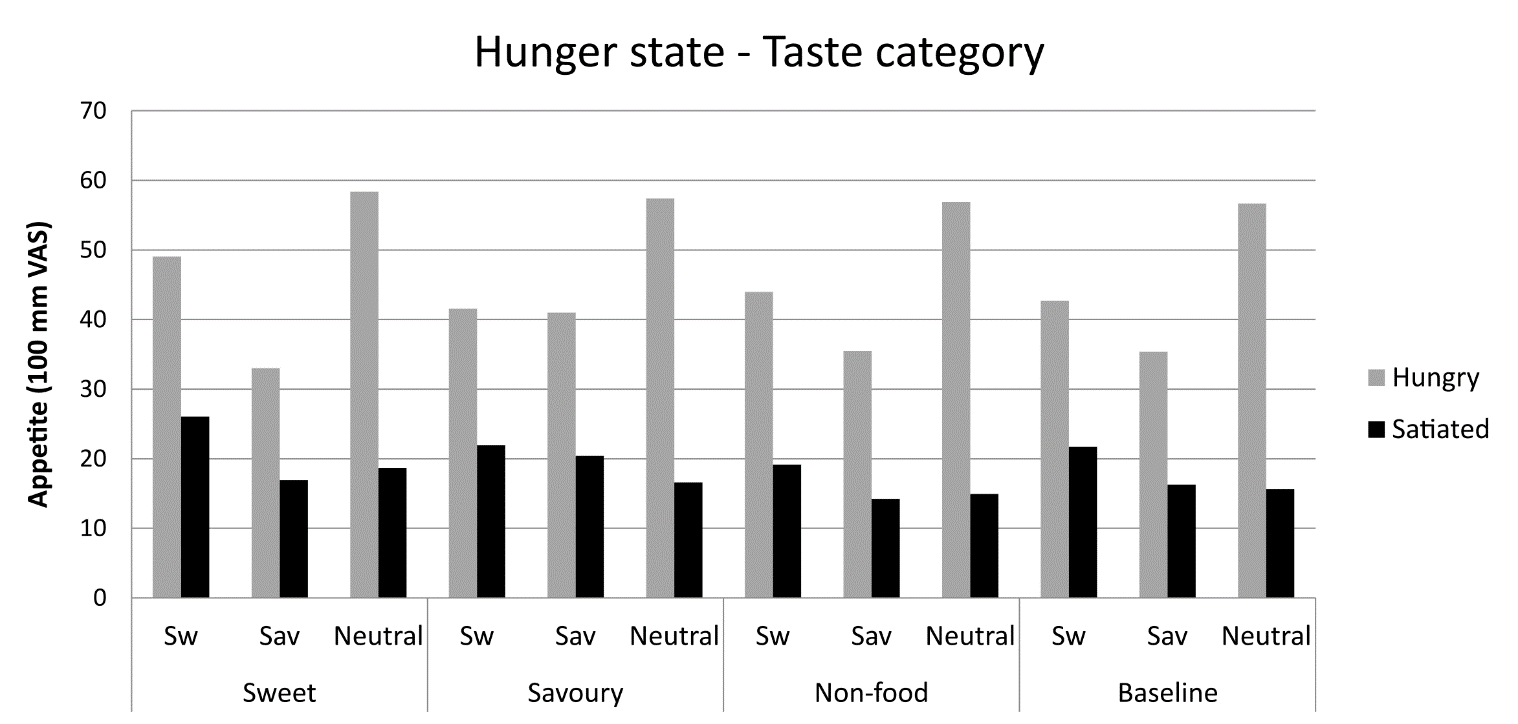


**Figure S1.** Raw appetite ratings for products from different taste categories: Sweet (Sw), savoury (Sav), neutral. Participants provided ratings in hungry and satiated states after exposure to odours from different categories: Sweet, savoury, non-food and baseline (no-odour).


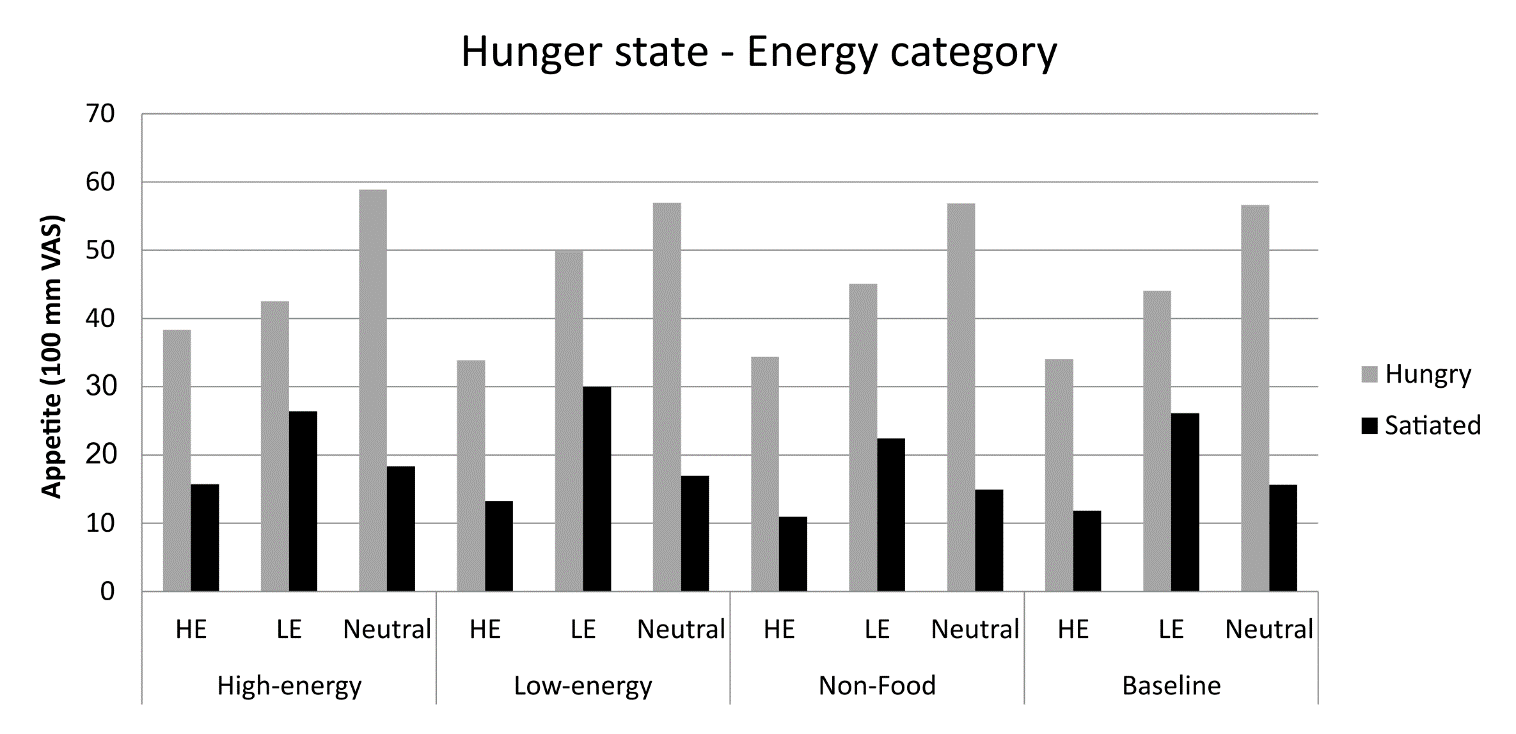


**Figure S2.** Raw appetite ratings for products from different energy categories: High-energy (HE), low-energy (LE), neutral. Participants provided ratings in hungry and satiated states after exposure to odours from different categories: high-energy, low-energy, non-food and baseline (no-odour).

© 2016 by the authors; licensee MDPI, Basel, Switzerland. This article is an open access article distributed under the terms and conditions of the Creative Commons by Attribution (CC-BY) license (http://creativecommons.org/licenses/by/4.0/).
